
JOURNAL INFORMATION
==============================
NLM Title Abbreviation: ScientificWorldJournal
Journal ID: ScientificWorldJournal
NLM Journal ID: 101131163
Journal ID: TSWJ

The Scientific World Journal

ISSN: 2356-6140
EISSN: 1537-744X
Publisher: Wiley

ARTICLE INFORMATION
==============================
PMCID: PMC3909732
PMID: 19618089
DOI: 10.1100/tsw.2009.88
Article ID: 541261
Article version: 2
Subjects: Directions in Science

Tumor-Suppressive microRNAs in Lung Cancer: Diagnostic and Therapeutic Opportunities

Sempere Lorenzo F. 1
Liu Xi 2
Dmitrovsky Ethan 1 2 3 *
1 Department of Medicine, Dartmouth-Hitchcock Medical Center, Lebanon, NH and Dartmouth Medical School, Hanover, NH, United States
2 Department of Pharmacology and Toxicology, Dartmouth-Hitchcock Medical Center, Lebanon, NH and Dartmouth Medical School, Hanover, NH, United States
3 Norris Cotton Cancer Center, Dartmouth-Hitchcock Medical Center, Lebanon, NH and Dartmouth Medical School, Hanover, NH, United States
*Ethan Dmitrovsky: ethan.dmitrovsky@dartmouth.eduAcademic Editor: Martin Goette

Publication date: 2009
Electronic publication date: 2009 Jul 13
Volume: 9
First page: 626
Last page: 628
Received 2009 Apr 13; Accepted 2009 Jun 29
Copyright: Copyright © 2009 Lorenzo F. Sempere et al.
Copyright year: 2009
License: This is an open access article distributed under the Creative Commons Attribution License, which permits unrestricted use, distribution, and reproduction in any medium, provided the original work is properly cited.
License URL: https://creativecommons.org/licenses/by/3.0/

Keywords: microRNA (miRNA, miR), lung cancer, expression profiling, in situ hybridization, miR-34c, miR-142-5p, miR145, cyclin E

Funding: National Cancer Institute RO1-CA087546 National Cancer Institute RO1-CA111422 National Cancer Institute RO3-CA130102 Susan G. Komen Breast Cancer Foundation PDF0503563

==============================
Directions in Science
*TheScientificWorldJOURNAL* (2009) 9, 626–628
ISSN 1537-744X; DOI 10.1100/tsw.2009.88

TheScientificWorldJOURNAL
www.thescientificworld.com

### Tumor-Suppressive microRNAs in Lung Cancer: Diagnostic and Therapeutic Opportunities

Lorenzo F. Sempere[1], Xi Liu[2], and Ethan Dmitrovsky[1,2,3,*]

*Department of [1]Medicine, [2]Pharmacology and Toxicology, and [3]Norris Cotton Cancer Center, Dartmouth-Hitchcock Medical Center, Lebanon, NH and Dartmouth Medical School, Hanover, NH*

**E-mail:** Lorenzo.F.Sempere@Dartmouth.EDU; Xi.Liu@Dartmouth.EDU; Ethan.Dmitrovsky@Dartmouth.EDU

*Received April 13, 2009; Accepted June 29, 2009; Published July 13, 2009*

**KEYWORDS:** microRNA (miRNA, miR), lung cancer, expression profiling, *in situ* hybridization, miR-34c, miR-142-5p, miR145, cyclin E

Lung cancer is the leading cause of cancer-related death for both men and women in the U.S. and other countries. In 2008, 215,020 new cases were diagnosed and a total of 161,840 patients died from lung cancer in the U.S.[1]. Despite a substantial decrease in active smokers, which would eventually result in a considerable reduction in its incidence, lung cancer will continue to be a major worldwide health-care problem for several decades. This will be due both to current smokers and the large number of former smokers who are at high risk for developing lung cancer. Lung cancer, as for many other cancer types, is not a single disease, but rather a collection of diseases that present different histopathological, molecular, and clinical characteristics. Improved diagnostic and therapeutic tools are needed to better manage and treat those patients afflicted with lung cancer.

In a recent study by Liu and colleagues published in *Clinical Cancer Research*, a subset of microRNAs were pinpointed as candidate tumor-suppressive species in lung cancer[2]. Using new mouse transgenic models for lung cancer, genome-wide microRNA expression analyses identified a signature of down-regulated microRNAs in lung cancers relative to adjacent normal lung tissues. This signature was validated in clinical specimens from lung cancer patients, underscoring the relevance of these microRNAs to this human malignancy. The authors hypothesized that functions of these down-regulated microRNAs were detrimental to normal cells and their loss in tumors was an active process contributing to uncontrolled growth of the affected cells. Consistent with this hypothesis, *in vitro* experiments demonstrated that individually restoring miR-34c, miR-145, or miR-142-5p expression markedly reduced proliferation of murine lung cancer cell lines.

The microRNAs are a recently discovered class of short, noncoding, regulatory RNAs that constitute a new and broad layer of gene regulation[3]. Binding of the ~18–25 nucleotide-long mature microRNAs to the 3'-untranslated region (UTR) of messenger RNAs (mRNAs) triggers translational down-regulation and/or increased degradation of the target gene products[3]. microRNA-mediated regulation of tumorigenesis has rapidly emerged as a promising area for translational cancer research. Altered expression of microRNAs has been linked to several cancer types, including lung cancer[4]. Moreover, altered tumor-suppressive and tumor-promoting functions of microRNAs have been shown to impact the course of tumorigenic processes greatly[5]. Thus, the potential clinical application of microRNAs as

*Corresponding author.
©2009 with author.
Published by TheScientificWorld; www.thescientificworld.com biomarkers and "druggable" targets is being intensely investigated in academic laboratories and pharmaceutical companies.

Liu and colleagues made use of novel mouse transgenic models for lung cancer driven by pulmonary epithelial-restricted expression of human wild-type or proteasome-resistant cyclin E; these mice developed lung adenocarcinomas[6]. Total RNA was independently extracted from adjacent normal and malignant lung tissues from these cyclin E–driven mouse models and hybridized against a locked nucleic acid (LNA) captured-probe microRNA microarray platform[7]. Differential expression of highlighted microRNAs was confirmed by quantitative real-time RT-PCR (qPCR) assays independently performed on RNA isolated from murine normal and malignant lung tissues. Notably, similar changes were observed in clinical specimens of different types of non–small cell lung cancers (NSCLC). These and other findings linked novel microRNAs to development of lung carcinogenesis.

To gain further insight into the spatial distribution of these highlighted microRNAs, *in situ* hybridization (ISH) assays based on LNA-modified probes were performed on formalin-fixed, paraffin-embedded sections[8]. miR-34c and miR-145 were detected in different cell types in normal murine lung and were repressed in lung adenocarcinomas. miR-34c accumulated in bronchial epithelial cells, whereas miR-145 accumulated in smooth muscle cells of lamina muscularis and blood vessels. Intriguingly, miR-34c expression, as determined by qPCR assays, was substantially down-regulated in human large cell carcinoma, squamous cell carcinomas, and adenocarcinomas, but not in bronchioalveolar carcinomas (BACs). BACs generally have a slower progression and a more favorable clinical prognosis than other types of NSCLC. Although retention of miR-34c expression in these BACs might simply reflect the cell type of origin of these lesions, it is tempting to speculate that miR-34c activity enables the functioning of tumor-suppressive pathways such as p53 (see below). In this context, detection of miR-34c expression by qPCR and/or ISH assays could be used as a clinically relevant prognostic marker.

The miR-34 family members (miR-34a, miR34b, and miR-34c) are transcriptionally regulated by p53 and act in concert to relay p53 tumor-suppressive signals by negatively inhibiting cell cycle progression via binding to mRNA of target genes: cyclin D1 and/or its kinase binding partners CDK4/6[9]. Liu and colleagues unraveled cyclin E as an additional target of miR-34c to halt cell proliferation. Notably, cyclin E is the very oncogene genetically engineered to drive tumorigenesis in these mouse models for lung cancer. Similarly, let-7 was demonstrated to be a potent negative regulator of K-ras in a K-ras-driven mouse model for lung cancer[10]. This reciprocal negative regulation between an oncogenic driver and tumor-suppressive microRNA might be disproportionately represented in these models. Nonetheless, this highlights the ability and/or requirement of neoplastic cells to disrupt microRNA-mediated, tumor-suppressive pathways as a key step to become and/or remain malignant. Given the etiological relevance of cyclin E alterations in lung cancer, functional studies should be extended to human cancer cell lines and to an even larger panel of clinical specimens in order to validate this functional interaction between miR-34c and cyclin E. In a separate study, miR-145 was shown to be under the transcriptional control of p53 and to act to repress expression of oncogenic c-myc in breast and colon cancer cell lines[11]. Thus, it will be important to identify the target genes of miR-145 and miR-142-5p to further understand their role in lung cancer.

Synthetic compounds that mimic or antagonize microRNA activities have been developed and shown to be efficacious in restoring or depleting miRNA function in *in vitro* cell systems. Moreover, promising results have been reported in mouse and nonhuman primates, where cholesterol levels can be regulated by intravenous injection of anti–miR-122, nucleotide-based compounds[12]. Although *in vivo* delivery of synthetic compounds that restore microRNA activities is a challenging task, there is reason to be optimistic that this will be accomplished. Restoration of tumor-suppressive microRNAs, such as miR-34c and miR-145, perhaps in combination with depletion of oncogenic microRNAs, would represent intriguing strategies to combat lung cancer.

### ACKNOWLEDGMENTS

This work was supported by National Institutes of Health (NIH) and National Cancer Institute (NCI) grants RO1-CA087546, (E.D.), RO1-CA111422 (E.D.), RO3-CA130102 (E.D.), a Samuel Waxman Cancer Research Foundation Award (E.D.), the American Lung Association (X.L.), a postdoctoral fellowship (PDF0503563) grant from the Susan G. Komen Breast Cancer Foundation (L.S.), a Hitchcock Foundation grant (L.S.), and an AACR-Pancreatic Cancer Action Network Award (L.S). Ethan Dmitrovsky is an American Cancer Society Clinical Research Professor supported by a generous gift from F.M. Kirby Foundation.

### REFERENCES

1. http://www.cancer.gov/cancertopics/types/lung
2. Liu, X., Sempere, L.F., Galimberti, F., Freemantle, S.J., Black, C., Dragnev, K.H., Ma, Y., Fiering, S., Memoli, V., Li, H., DiRenzo, J., Korc, M., Cole, C.N., Bak, M., Kauppinen, S., and Dmitrovsky, E. (2009) Uncovering growth-suppressive microRNAs in lung cancer. *Clin. Cancer Res.* **15**, 1177–1183.
3. Carthew, R.W. and Sontheimer, E.J. (2009) Origins and mechanisms of miRNAs and siRNAs. *Cell* **136**, 642–655.
4. Barbarotto, E., Schmittgen, T.D., and Calin, G.A. (2008) MicroRNAs and cancer: profile, profile, profile. *Int. J. Cancer* **122**, 969–977.
5. Ventura, A. and Jacks, T. (2009) MicroRNAs and cancer: short RNAs go a long way. *Cell* **136**, 586–591.
6. Ma, Y., Fiering, S., Black, C., Liu, X., Yuan, Z., Memoli, V.A., Robbins, D.J., Bentley, H.A., Tsongalis, G.J., Demidenko, E., Freemantle, S.J., and Dmitrovsky, E. (2007) Transgenic cyclin E triggers dysplasia and multiple pulmonary adenocarcinomas. *Proc. Natl. Acad. Sci. U. S. A.* **104**, 4089–4094.
7. Kauppinen, S., Vester, B., and Wengel, J. (2006) Locked nucleic acid: high-affinity targeting of complementary RNA for RNomics. *Handb. Exp. Pharmacol.* **(173)**, 405–422.
8. Sempere, L.F., Christensen, M., Silahtaroglu, A., Bak, M., Heath, C.V., Schwartz, G., Wells, W., Kauppinen, S., and Cole, C.N. (2007) Altered microRNA expression confined to specific epithelial cell subpopulations in breast cancer. *Cancer Res.* **67**, 11612–11620.
9. He, L., He, X., Lowe, S.W., and Hannon, G.J. (2007) microRNAs join the p53 network--another piece in the tumour-suppression puzzle. *Nat. Rev. Cancer* **7**, 819–822.
10. Kumar, M.S., Erkeland, S.J., Pester, R.E., Chen, C.Y., Ebert, M.S., Sharp, P.A., and Jacks, T. (2008) Suppression of non-small cell lung tumor development by the let-7 microRNA family. *Proc. Natl. Acad. Sci. U. S. A.* **105**, 3903–3908.
11. Sachdeva, M., Zhu, S., Wu, F., Wu, H., Walia, V., Kumar, S., Elble, R., Watabe, K., and Mo, Y.Y. (2009) p53 represses c-Myc through induction of the tumor suppressor miR-145. *Proc. Natl. Acad. Sci. U. S. A.* **106**, 3207–3212.
12. Petri, A., Lindow, M., and Kauppinen, S. (2009) MicroRNA silencing in primates: towards development of novel therapeutics. *Cancer Res.* **69**, 393–395.

**This article should be cited as follows:**

Sempere, L.F., Liu, X., and Dmitrovsky, E. (2009) Tumor-suppressive microRNAs in lung cancer: diagnostic and therapeutic opportunities. *TheScientificWorldJOURNAL* **9**, 626–628. DOI 10.1100/tsw.2009.88.
